# Duloxetine in treatment of refractory chronic tennis elbow: Two case reports

**DOI:** 10.1186/1752-1947-2-305

**Published:** 2008-09-17

**Authors:** Zaid Ahmad Wani, Shabir Ahmad Dhar, Mohammad Farooq Butt, Yasir Hassan Rather, Shano Sheikh

**Affiliations:** 1 Department of Psychiatry, Government Psychiatric Diseases Hospital, GMC Srinagar, Kashmir, J&K, 190010, India; 2 Department of Orthopaedics, GMC Srinagar, Kashmir, J&K, 190010, India; 3 Department of Psychology, Jamia Milia Islamia, Jamia Nagar, New Delhi, 110025, India

## Abstract

**Introduction:**

Tennis elbow is a common musculoskeletal disorder; management options include physiotherapeutic, medical, surgical, and other forms of intervention. Some patients remain symptomatic despite best efforts. We present two patients who did not respond to medical and surgical treatments, and whose symptoms were relieved with duloxetine. This is the first report on the use of duloxetine to treat tennis elbow.

**Case presentation:**

Two mentally healthy young Asian women aged 32 and 27 years, each with tennis elbow of about 18 months duration continued to suffer pain despite treatment with analgesics, local steroid injections, physiotherapy, cryotherapy, ultrasound, and surgical release, among other interventions. Both showed substantial improvement within 4 to 6 weeks of receiving monotherapy with duloxetine 60 mg/day. Both were pain-free with continued treatment at a 6-month follow-up.

**Conclusion:**

Duloxetine may be a useful treatment option in patients with chronic tennis elbow, even those who have failed conventional medical, physiotherapeutic, surgical, and other forms of management.

## Introduction

Lateral epicondylalgia or tennis elbow is a common cause of pain and disability; it often develops in non-athletes. It is characterized by pain and tenderness centered around the lateral epicondyle. The source of the pain was initially thought to be due to extensor carpi radialis brevis degeneration. However, it is now recognized that the lateral epicondyle, the annular ligament, the radial head and the capitellum may also contribute to the experience of pain in tennis elbow [1].

Several factors have been implicated in the causation of tennis elbow. These include overuse of the affected limb, repetitive forceful movements, training errors, misalignments, flexibility problems, ageing, poor circulation, strength deficits and muscle imbalances [2,3].

Tennis elbow can be difficult to treat. The condition is prone to recurrence, and the symptoms may last for several weeks or months, with the average duration of a typical episode reported to be between 6 months and 2 years [4]. Non-operative treatment is successful in effecting a resolution of symptoms in most patients. Such conservative treatment options include analgesics, tennis elbow support, ultrasonic therapy, and splint immobilization. Local corticosteroid injections, local autologous blood infiltration, laser therapy, and nitrogen cryotherapy have been used as semi-conservative methods [4,5]. All of these aim at reducing the pain and improving the functional status of the affected limb. Surgery is offered to resistant cases; outcomes are not always successful [6].

Duloxetine is an antidepressant which acts by serotonin and noradrenalin reuptake inhibition [7]. Duloxetine has been found to be helpful in patients with painful diabetic neuropathy [8] and fibromyalgia [9]. We hypothesized that duloxetine may benefit two patients who were referred to us for the management of chronic, refractory tennis elbow. To the best of our knowledge, this is the first report on the use of duloxetine to treat tennis elbow.

## Case presentation 1

A 32-year-old woman, presented with symptoms of tennis elbow of the right limb of 18 months duration. She had been treated with analgesics, splint immobilization and rest, local steroid infiltrations, ultrasonic therapy and autologous blood infiltration over a period of 1 year. She had also undergone lateral tendon release 7 months earlier. A psychiatric evaluation was sought because of the unrelenting nature of the pain arising from the common extensor muscles of the forearm. Physical examination showed tenderness just distal and anterior to the lateral epicondyle along with pain with resisted wrist extension with elbow in full extension. Clinical interview identified no Axis I psychiatric disorder. The pain on the visual analogue scale (VAS) was about 70–75 mm, with 0 being none and 100 being maximum pain. The initial Nirschl stage was 5 [10].

She was prescribed duloxetine in a dose that was increased to 60 mg per day over 5 days; analgesic treatment was stopped. She reported a gradual reduction in pain; VAS scores dropped to 40 after 3 weeks, and to 25 after 4 weeks. She was substantially pain-free after 6 weeks of treatment. The tests for elbow tendinosis, including resisted wrist extension and supination and 3rd digit extension, did not produce pain. The Nirschl stage was 2.

## Case presentation 2

A 27-year-old woman was diagnosed with right-sided tennis elbow of 18 months duration. She had been treated with analgesics, plaster immobilization, multiple steroid infiltrations, cryotherapy, ultrasonic therapy, and prolonged brace wear. She had experienced limited pain relief lasting for less than a fortnight on most occasions. Nirschl tendon release was done after the conservative methods failed. She experienced partial pain relief for 4 weeks but again developed pain. She was referred for a psychiatric evaluation but this did not reveal any Axis I psychiatric disorder. The pain on VAS was 65–70 mm with a Nirschl stage of 4.

She was prescribed duloxetine 60 mg/day. After 4 weeks, the VAS score had dropped to 30. The tests for elbow tendinosis, including resisted wrist extension and supination and third digit extension, did not produce pain. The Nirschl stage was 2.

Both patients continued with duloxetine to a 6-month follow-up, at which time VAS scores were recorded as zero.

## Discussion

Chronic pain compromises quality-of-life and impairs work performance. While a cure may not be feasible, treatment efforts should aim for pain relief and improvement in the quality-of-life.

Pain is transmitted from peripheral sites along two sets of afferent nerves, that is, the A delta and C fibers. These synapse in the dorsal horn of the spinal cord. Preliminary processing of pain information occurs here before transmission through ascending tracts to the thalamus and higher brain centers. Pain information however, can be modulated by the activity of descending inhibitory fibers passing from the brain to the spinal cord. The neurotransmitters primarily involved in the descending pathways, that is, norepinephrine and serotonin, act synergistically in reducing the transmission of pain information from the periphery to the central nervous system [11,12]. Analgesia produced by antidepressants is thought to be mediated by enhanced activity of norepinephrine and serotonin in descending fibers [13]. Some antidepressants also inhibit sodium-channel function, which can dampen the activity of pain-relaying neurons [14]. Enhanced activity of norepinephrine and serotonin in descending fibers as a result of duloxetine administration could have resulted in the improvements observed in the two cases reported. We speculate that these effects raise the pain threshold in the affected patients, leading to a decreased perception of pain. We are, however, unable to explain the maintained state of recovery of both patients, which must have involved a reversal of the pathology locally.

## Conclusion

Duloxetine may relieve pain in patients with chronic tennis elbow, including those who have failed to respond to medical, physiotherapeutic, surgical, and other interventions. The benefits of duloxetine and its impact on the pathology of the disorder merit investigation in prospective clinical trials.

## Consent

Written informed consent was obtained from both patients for publication of these case reports. Copies of the written consent are available for review by the Editor-in-Chief of this journal.

## Competing interests

The authors declare that they have no competing interests.

## Authors' contributions

ZAW reviewed the literature, and conceived of and drafted the manuscript; YHR performed the psychiatric assessment of the patients including the follow-up. SAD and MFB were responsible for the orthopedic assessment and revision of the paper. SS performed the psychological assessment of the patients. All authors read and approved the final manuscript.
